# Observation of re-entrant spin reorientation in TbFe_1−*x*_Mn_*x*_O_3_

**DOI:** 10.1038/srep33448

**Published:** 2016-09-16

**Authors:** Yifei Fang, Ya Yang, Xinzhi Liu, Jian Kang, Lijie Hao, Xiping Chen, Lei Xie, Guangai Sun, Venkatesh Chandragiri, Chin-Wei Wang, Yiming Cao, Fei Chen, Yuntao Liu, Dongfeng Chen, Shixun Cao, Chengtian Lin, Wei Ren, Jincang Zhang

**Affiliations:** 1 Materials Genome Institute, Shanghai University, Shanghai 200444, China; 2 Department of Physic, Shanghai University, Shanghai 200444, China; 3 Neutron Scattering Laboratory, China Institute of Atomic Energy, Beijing 102413, China; 4 Insititute of Nuclear Physics and Chemistry, China Academy of Engineering Physics, Mianyang 621999, China; 5 ANSTO, Kirrawee Dc, NSW 2232, Australia; 6 Max Planck Institute for Solid State Research, Heisenbergstraße 1, D-70569 Stuttgart, Germany

## Abstract

We report a spin reorientation from Γ_4_(*G*_*x*_, *A*_*y*_, *F*_*z*_) to Γ_1_(*A*_*x*_, *G*_*y*_, *C*_*z*_) magnetic configuration near room temperature and a re-entrant transition from Γ_1_(*A*_*x*_, *G*_*y*_, *C*_*z*_) to Γ_4_(*G*_*x*_, *A*_*y*_, *F*_*z*_) at low temperature in TbFe_1−*x*_Mn_*x*_O_3_ single crystals by performing both magnetization and neutron diffraction measurements. The Γ_4_ − Γ_1_ spin reorientation temperature can be enhanced to room temperature when *x* is around 0.5 ~ 0.6. These new transitions are distinct from the well-known Γ_4_ − Γ_2_ transition observed in TbFeO_3_, and the sinusoidal antiferromagnetism to complex spiral magnetism transition observed in multiferroic TbMnO_3_. We further study the evolution of magnetic entropy change (−ΔS_M_) *versus* Mn concentration to reveal the mechanism of the re-entrant spin reorientation behavior and the complex magnetic phase at low temperature. The variation of −ΔS_M_ between *a* and *c* axes indicates the significant change of magnetocrystalline anisotropy energy in the TbFe_1−*x*_Mn_*x*_O_3_ system. Furthermore, as Jahn-Teller inactive Fe^3+^ ions coexist with Jahn-Teller active Mn^3+^ ions, various anisotropy interactions, compete with each other, giving rise to a rich magnetic phase diagram. The large magnetocaloric effect reveals that the studied material could be a potential magnetic refrigerant. These findings expand our knowledge of spin reorientation phenomena and offer the alternative realization of spin-switching devices at room temperature in the rare-earth orthoferrites.

The rare-earth orthoferrites RFeO_3_ (R = rare-earth elements) is a family of functional materials with large magnetoelectric (ME) coupling and optomagnetic properties^1^,^2^. Some of RFeO_3_ have been reported to be multiferroic materials with possible applications^3^,^4^,^5^. Furthermore, spin reorientation phase transition in such antiferromagnetic (AFM) insulators has attracted much attention since high-temperature Γ_4_(*G*_*x*_, *A*_*y*_, *F*_*z*_) phase usually transforms to Γ_2_(*F*_*x*_, *C*_*y*_, *G*_*z*_) at lower temperature^3^. Meanwhile, the rare-earth manganites RMnO_3_, aroused great interest in spintronics due to both colossal magnetoresistance (CMR) and magnetoelectric coupling effects^6^,^7^,^8^,^9^,^10^,^11^. The neutron diffraction experiments have revealed the existence of helical spin structure in RMnO_3_ (R = Tb, Dy) systems^12^,^13^,^14^,^15^ being the origin of their magnetoelectric coupling effect^16^,^17^,^18^,^19^.

The compounds of TbMnO_3_ and TbFeO_3_ both belong to the orthorhombic space group *Pbnm* with the same distorted perovskite structure. But they show distinct magnetic properties due to their totally different spin configurations. TbFeO_3_ has a canted AFM spin ordering caused by the Dzyaloshinskii-Moriya (DM) interaction^5^. Previous reports confirmed that most RFeO_3_ (R = Tb, Er, Sm, Tm, Yr, Sc, Nd, etc.) should undergo a spin reorientation transition from Γ_4_ to Γ_2_^3^,^5^,^20^,^21^,^22^,^23^,^24^,^25^,^26^,^27^,^28^, as a second-order magnetic phase transition. Exceptionally, DyFeO_3_ exhibits an interesting Γ_4_ → Γ_1_(*A*_*x*_, *G*_*y*_, *C*_*z*_) phase transition which completely annihilates weak magnetic moments observed in Γ_4_(*F*_*z*_) or Γ_2_(*F*_*x*_), along any crystallographic direction. This phenomenon has been observed in magnetization measurement, but not yet detected by neutron diffraction experiment due to the high absorption of Dy element. On the other hand, TbMnO_3_ manifests both magnetoelectric and magnetocaloric effect^29^,^30^, the latter is a key ingredient for high efficient magnetic refrigerant with large magnetic entropy change (−Δ*S*_*M*_). It was previously shown that the spiral spin structures of Mn^3+^ ions below 27 K lead to ferroelectricity^29^ and mutual controls of magnetism and electricity^14^,^31^. However, the spiral spin order is weak and can be easily destroyed by doping transition metals. Thus, although the parent compounds without doping are well investigated, the rich physics due to off-stoichiometry TbFe_1−*x*_Mn_*x*_O_3_ is largely unexplored. It is our goal of present work to find out new magnetic phase and reveal the mechanism of new spin reorientation in TbFe_1−*x*_Mn_*x*_O_3_ system, from which some remarkable behaviors expected to be found due to competitive magnetic phases that do not exist in both TbMnO_3_ and TbFeO_3_.

In this work, we synthesized a series of TbFe_1−*x*_Mn_*x*_O_3_ and reported their special magnetic phase transitions by performing magnetization and neutron powder diffraction (NPD) measurements. We demonstrate that the phase transition of Γ_4_ → Γ_1_ → Γ_4_ exists in TbFe_0.75_Mn_0.25_O_3_ single crystal, rather than the common transition of Γ_4_ → Γ_2_ as observed in TbFeO_3_ and other orthoferrites. From a practical point of view, Γ_4_ → Γ_1_ (weak magnetic moment to zero net moment) transition may find use even with easily-obtained polycrystalline samples whereas we need to grow single crystals to observe Γ_4_ → Γ_2_ (weak magnetic moment along *c* to *a* direction) transition. The magnitude of the magnetocaloric effect is found to be large and strictly resembles the observed magnetic features. The evolution of entropy change *versus* Mn doping are presented and discussed with in the scenario of Mn substitution-induced anisotropic interaction.

## Results

### Magnetometry and neutron diffraction measurements

**Figure 1:**
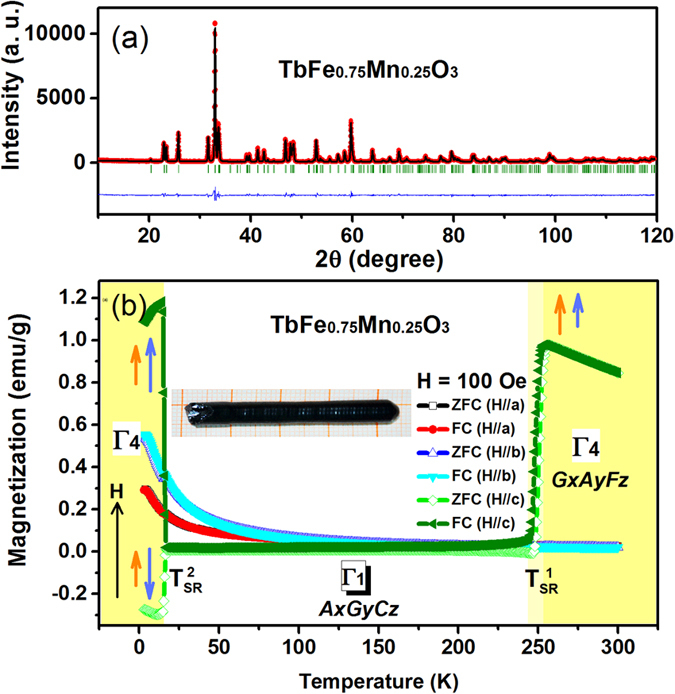
Powder XRD refinement and magnetic phase transition of TbMn_0.25_Fe_0.75_O_3_. (**a**) XRD patterns obtained by the ground crystal powders at room temperature. Inset is the optical-floating-zone grown single crystal on a grid of millimeter. (**b**) The temperature dependence of magnetization curves under *H* = 100 Oe. The shaded parts denote the magnetic phase transition and divarication of ZFC/FC. The arrows show the evolution of magnetization arising from Tb (blue) and Fe/Mn (orange).

The x-ray diffraction patterns for the TbFe_1−*x*_Mn_*x*_O_3_ with *x* = 0.25 are plotted in Fig. 1(a). The Rietveld refinement results show that the sample has a distorted orthorhombic perovskite structure (*Pbnm*) and no additional phases are identified. Figure 1(b) shows its temperature dependence of ZFC and FC magnetization curves with *H* = 100 Oe along the *a (H *|| *a*), *b (H* || *b*) and *c (H* || *c*) directions, denoted as 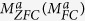, 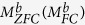, and 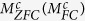. The total magnetic moment is parallel to *c* axis between 254 and 300 K. A sharp drop in 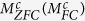 occurs between 254 and 245 K, signaling the spin reorientation transition of Fe^3+^ ions^23^. When the temperature is between 16 and 254 K, the sample shows an antiferromagnetic state for *H* || *c*, while both 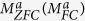 and 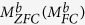 increase slowly for *H* || *a* and *H* || *b* with the decreasing temperature. This resembles the phase transition of Γ_4_ → Γ_1_ spin reorientation at *T*_*SR*_ = 254 K. Thus, we speculate that the magnetic structure transforms from the canted antiferromagnetism with weak ferromagnetism along the *c* axis (*G*_*x*_, *A*_*y*_, *F*_*z*_) to the major G-type antiferromagnetic vector along the *b* axis (*A*_*x*_, *G*_*y*_, *C*_*z*_). In this case, there is no net magnetic moment along the *c* axis in the wide range of temperature between 16 and 254 K. Interestingly, as temperature decreases below 16 K, the magnetic moment along the *c* axis turns to be negative with a possible (*G*_*x*_, *A*_*y*_, *F*_*z*_) configuration in negative magnetization state. The moment configuration speculated from the magnetometry is shown as the arrows in Fig. 1(b). The arrows show the evolution of magnetization arising from Tb (blue arrows) and Fe/Mn (orange arrows), respectively. The net moments of Tb and Fe tend to keep aligning along *c*-axis and parallel to the direction of applied field, which is responsible for the weak ferromagnetism above 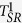. Nevertheless, due to the *d* − *f* interactions of Fe/Mn and Tb ions, the larger net moment of Tb ions become antiparallel to those of Fe ions and the direction for applied field below 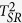 in ZFC mode, leading to a negative magnetization state. However, in FC mode, both the net moments of Tb and Fe ions keep parallel to the direction of applied field, resulting in large net magnetization below 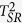.

**Figure 2:**
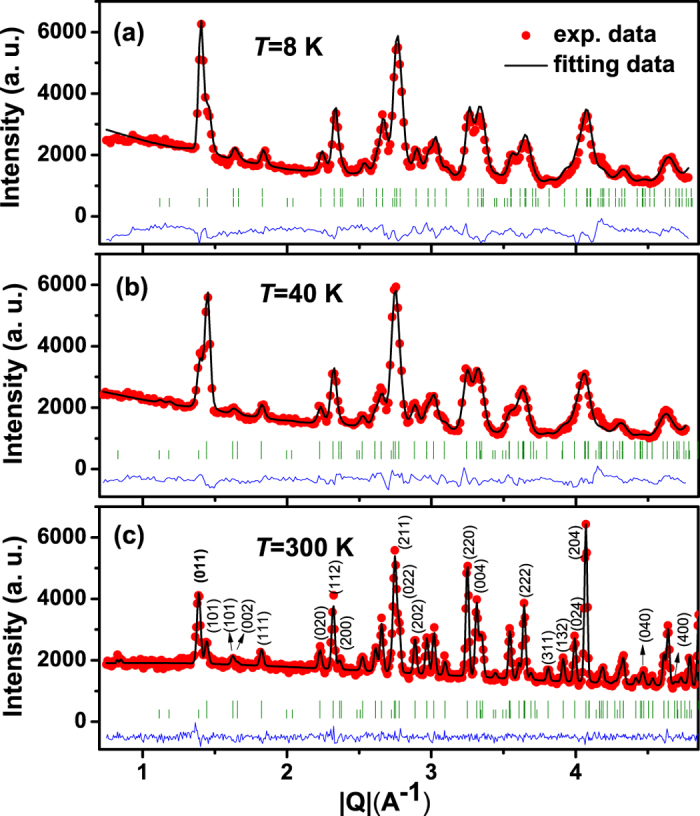
NPD patterns for TbFe_1−*x*_Mn_*x*_O_3_ with *x* = 0.25 sample, obtained at (**a**) *T* = 8 K, (**b**) *T* = 40 K and (**c**) *T* = 300 K, respectively. The two lines of vertical bars present Bragg peaks for the crystal structures and the crystal with magnetic structures of Fe^3+^/Mn^3+^ respectively.

To confirm our speculation on the nature of the intriguing spin reorientation transition phenomena, the NPD experiments were performed and the results are shown in Fig. 2(a–c) for *T* = 8, 40, and 300 K, respectively. All the structural Bragg peaks show no position shift or split, indicating the absence of structural phase transition at the different temperatures. Symmetry analysis was performed based on the crystal structure of TbFe_1−*x*_Mn_*x*_O_3_ (*x* = 0.25), which is an orthorhombically-distorted perovskite structure with space group *Pbnm*. The lattice parameters for this crystal structure are *a* = 5.284 Å, *b* = 5.603 Å and *c* = 7.530 Å. For our orthoferrites, the *k* = 0 propagation vector was adopted as usual. According to the symmetry theory proposed by White^32^ and Bertaut^20^, Γ_5_ and Γ_8_ are incompatible with a net moment on the iron sites. Γ_3_ is not consistent with the observed strong antiferromagnetic coupling between nearest iron neighbors. So Γ_1_, Γ_2_ and Γ_4_ could be possible magnetic structure for this compound.

**Figure 3:**
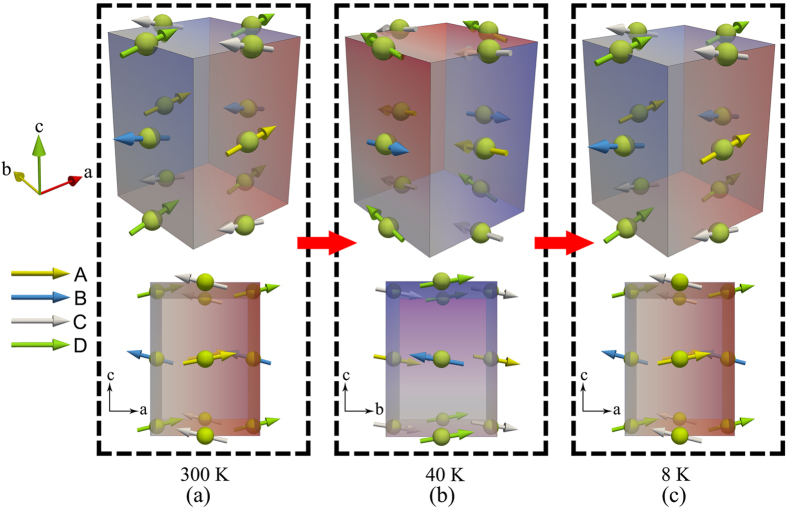
Evolution of magnetic phases for TbFe_0.75_Mn_0.25_O_3_. The upper panels display (**a**) Γ_4_(*G*_*x*_, *A*_*y*_, *F*_*z*_) phase at *T* = 300 K, (**b**) Γ_1_(*A*_*x*_, *G*_*y*_, *C*_*z*_) phase at *T* = 40 K, and (**c**) Γ_4_(*G*_*x*_, *A*_*y*_, *F*_*z*_) phase at *T* = 8 K. The lower panels illustrates the side view of the upper panels, respectively. Arrows of A-D represent four kinds of spin moments of Fe(Mn) ions.

Then Rietveld refinements were performed to test these possibilities with the orthorhombic *Pbnm* structure. The results show that the data obtained at 8 and 300 K fit well with the magnetic structures of Γ_4_(*G*_*x*_, *A*_*y*_, *F*_*z*_), and the data obtained at 40 K fit well with Γ_1_(*A*_*x*_, *G*_*y*_, *C*_*z*_). The derived structures of Fe/Mn are schematically drawn in Fig. 3(a–c), respectively. Arrows of A-D represent four types of location of Fe(Mn) ions, and the corresponding refined magnetic moments along different crystallographic axes are given in supplemental material^33^. Figure 3(a) illustrates an orthorhombic perovskite with Fe/Mn having G-type AFM spin order along the *a* axis, A-type AFM spin order along the *b* axis, and F-type FM spin order along the *c* axis, consistent to the (*G*_*x*_*A*_*y*_*F*_*z*_) configuration at room temperature in Bertaut’s notation^20^. This type of commensurate spin order is observed to decline at 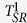 = 254 K and totally collapse at 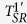 = 245 K, resulting in a new antiferromagnetic phase with no net magnetization along any direction^32^. This intriguing magnetic phase configuration is found to be *A*_*x*_*G*_*y*_*C*_*z*_ as shown in Fig. 3(b), instead of the common *F*_*x*_*C*_*y*_*G*_*z*_ reported for most RFeO_3_ systems. This transition at 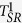 is characterized by the relative change of the (011) intensity and the (101) magnetic Bragg peaks around |Q| = 1.4A^−1^ as shown in Fig. 2(b,c), implying that the moments of Fe^3+^ rotate from the *a* to *b* axis upon cooling as indicated in Fig. 3(a–c). With the further decrease of temperature, both 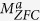
(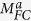) and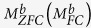 increase gradually. It is noted that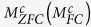 remains vanished till 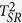 = 16 K, then a sudden increase arises at 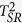. This sharp transition from Γ_1_ back to Γ_4_ is accomplished within 1 K and the results are confirmed by NPD. The ordering of Tb^3+^ has not been observed at T ≥ 8 K in our present measurement and further research at lower temperature is required. The 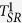 at 254 K is believed to be driven by both Fe-Fe and Tb-Fe/Mn sub-lattice interactions, while 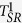 at 16 K arises from the enhanced interactions of Tb-Fe/Mn sub-lattice^5^.

### Spin reorientation at 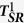


**Figure 4:**
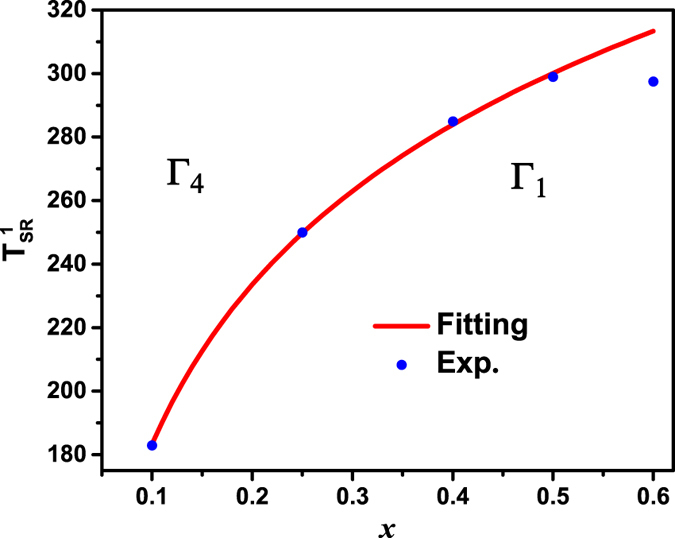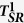 **as a function of** ***x*** in TbFe_1−*x*_Mn_*x*_O_3_ system. The solid line is the fitting of Eq. (1).

Since Γ_4_ configuration is characterized by the net moments along the *c* axis, the magnetic phase transition of Γ_4_ → Γ_1_ may undergo a transformation of the Fe^3+^ sublattice from weak ferromagnetism to complete antiferromagnetism upon the decreasing temperature. In order to reveal the relationship between Mn substitution and the changes in the anisotropy fields on the sublattices, a formula is developed by Holmes *et al.*^23^. Based on molecular field theory, the doping concentration dependence of 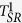 obeys the following equation for *x *≥ *x*_*c*_.


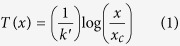


where *k*′ is a positive constant related to the second-order anisotropy fields in the *b-a*, *b-c*, *c-a* planes. *x*_*c*_ characterizes a critical doping concentration from a hypothesis that at *x* = *x*_*c*_, the Γ_4_ → Γ_1_ spin reorientation first appears at *T* = 0 K. This formula reveals that Mn substitutions can be used to shift the Γ_4_ → Γ_1_ spin reorientation to be near or above room temperature. Figure 4 shows the experimental and fitting results for TbFe_1−*x*_Mn_*x*_O_3_. Corresponding Eq. (1) with *x* = 0 ~ 0.6 and parameters *x*_*c*_ ≈ 0.0080 and k′ = 0.0060 is adopted for this system. Since the Eq. (1) is only valid at low doping concentration, we can obtain good fitting up to *x* = 0.5. As shown in Fig. 4, 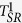 could be taken as the onset point of the first spin reorientation transition, and 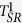 can be enhanced to 299 K at *x* = 0.5. For *x* > 0.6, the spin reorientation transition phenomenon was not observed up to their Néel temperatures (*T*_N_). Moreover, the *T*_N_ in Mn-rich TbFe_1−*x*_Mn_*x*_O_3_ becomes much lower than that of the TbFeO_3_ (*T*_N_ = 650 K), since Mn doping could weaken both the Tb^3+^-Fe^3+^ interaction and the Fe^3+^-Fe^3+^ interaction. In the high temperature Γ_4_ region of TbFe_1−*x*_Mn_x_O_3_, the Tb^3+^-Fe^3+^ interaction is much stronger than that of Fe^3+^-Fe^3+^ interaction and the former one causes the parallel aligns between the moments of Tb^3+^ and Fe^3+^ to give rise to the net moments along *c* axis^5^. This scenario is supported from the magnetization behavior along *c* axis as indicated in Fig. 1(b). As a consequence, the substitution of Mn for Fe breaks the original Tb^3+^-Fe^3+^ interaction, leading to the shift of 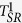 towards higher temeprature^34^.

### Spin reorientation at 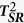


**Figure 5:**
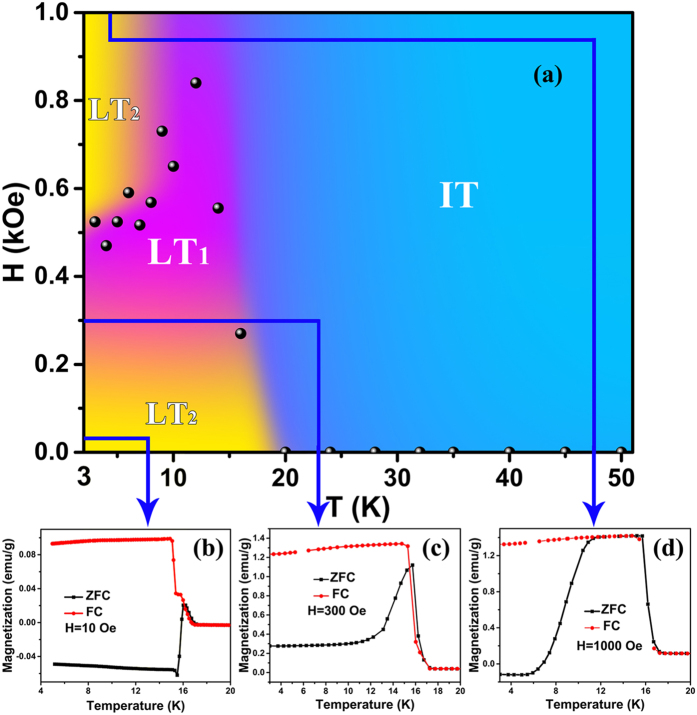
The phase diagram of magnetization vectors of TbFe_1−*x*_Mn_*x*_O_3_ (*x* = 0.25) by tuning the applied magnetic field and temperature. LT_1_: low temperature phase-1, LT_2_: low temperature phase-2, IT: intermediate temperature phase.

Compare with the Γ_4_ → Γ_1_ spin reorientation at 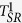, the Γ_1_ → Γ_4_ spin reorientation at 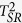 presents more complex magnetic phase since the ordering degree and interaction between magnetic ions get larger and stronger at low temperature. We herein take TbFe_0.75_Mn_0.25_O_3_ as a representative of TbFe_1−*x*_Mn_*x*_O_3_ system to discuss the characteristic of re-entrant Γ_1_ → Γ_4_ spin reorientation. Figure 5(a) shows a phase diagram of magnetic field *versus* temperature for *x* = 0.25, and the data points of crossover field *H*_*cro*_ were obtained from the isothermal *M*-*H* curves, in which *H*_*cro*_ is taken as the cross point between *M*^*a*^-*H* and *M*^*c*^-*H* curves (i.e. when *M*^*a*^ = *M*^*c*^ at a given temperature) from *H* = 0 kOe to 70 kOe. As the applied field increases, the M−T curves of the same system show dramatically different characteristics. The solid data points at the phase boundaries represent the second spin reorientation transition temperature 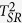 for *x* = 0.25 sample, which divides the diagram into three magnetic phases, i.e., the first low temperature phase (LT_1_), the second low temperature phase (LT_2_), and intermediate temperature phase (IT). Both LT_1_ and LT_2_ phases are characterized as Γ_4_ type, and IT is of Γ_1_ type. LT_1_ presents a weak ferromagnetic phase with Γ_4_ type while LT_2_ regions show the negative magnetization behavior with Γ_4_ type. This phase diagram illustrates the phase transition Γ_1_ → Γ_4_ can be modified by the external field at low temperature, resulting in rich variation of magnetocrystalline anisotropy.

## Discussion

Several factors may affect the spin reorientation transition phenomena: single ion anisotropy, DM interaction, exchange interaction, and magnetic anisotropy^35^. For a rare-earth ion, 4*f* orbital electrons make it special in bonding and the compounds have large single ion anisotropy. Consequently, the giant magnetocrystalline anisotropy and sharp spin reorientation can be attributed to the single ion anisotropy of Tb^3+^ with large angular momentum. For a spin-canted system^36^,^37^,^38^,^39^, a ubiquitous antisymmetric interaction **D** ⋅ (**S**_1_ × **S**_2_) exists, which is linear with respect to the spin-orbit coupling and exchange interaction. The magnitude of **D** can be expressed roughly as *D* ≈ (Δ*g*)/(*g*)*J*_*super*_, where *g* is the gyromagnetic ratio, Δ*g* is its deviation from the value for a free electron, and *J*_*super*_ is the strength of superexchange interaction. According to the magnetocaloric and NPD data, we can regard the magnetic entropy change between the *a* and *c* axes as a measurement of magnetocrystalline anisotropy energy and analyze the superexchange interactions in our TbFe_1−*x*_Mn_*x*_O_3_ system.

In order to illustrate the variation of magnetocrystalline anisotropy energy *versus* Mn doping concentration, we estimate that the magnetic entropy change (−Δ*S*_*M*_, where 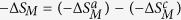) between the *a* and *c* axes in TbFe_1−*x*_Mn_*x*_O_3_ single crystals. According to Maxwell’s relation^40^,^41^, the magnetic entropy change in a thermodynamic process can be estimated using the following equation


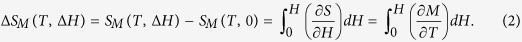


**Figure 6:**
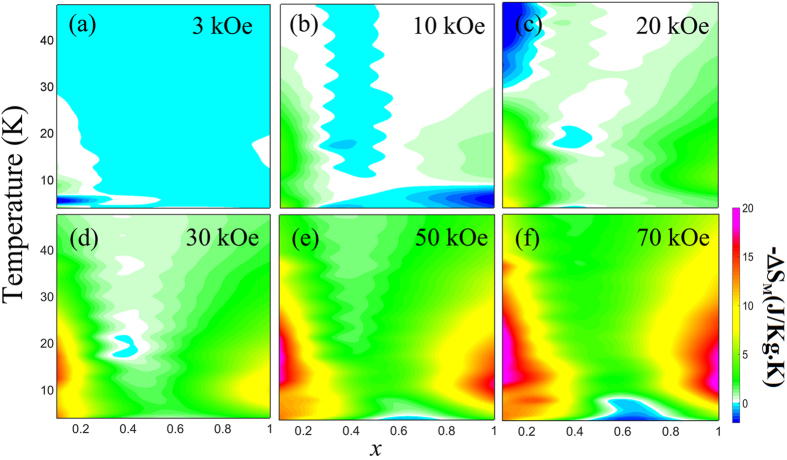
The distribution of magnetic entropy change as a function of the temperature and Mn concentration ***x*** under different applied fields. (**a**) *H* = 3 kOe, (**b**) *H* = 10 kOe, (**c**) *H* = 20 kOe, (**d**) *H* = 30 kOe, (**e**) *H* = 50 kOe, (**f** ) *H* = 70 kOe.

We take the Δ*T* = 1 K (or no more than 5 K at higher temperature) and Δ*H* = 1 kOe and the computed results are illustrated in Fig. 6(a–f). From Eq. (2), the magnetic entropy change is a function of both temperature and magnetic field. Therefore, by changing temperature and magnetic field, the direction of magnetization vector will rotate due to the magnetocrystalline anisotropy field, and the distribution of anisotropy energy will also vary. Hereafter, we denote the magnetocrystalline anisotropy energy as *E*_ani_. It is noted that the −Δ*S*_*M*_ value decreases from *x* = 0 to *x* = 0.5 and then increases to *x* = 1, suggesting a similar tendency for the *E*_ani_. The evolution of −Δ*S*_*M*_ *versus* doping concentration *x* implies that the superexchange interaction weakens along the *c* axis while enhances along the *a* or *b* axes for 0 ≤ *x* ≤ 0.5, and the reversed case holds for 0.5 ≤ *x* ≤ 1.

In TbFe_0.75_Mn_0.25_O_3_, the hard axis is along the *c* direction, and the easy magnetization vector is along *a* direction as shown in Fig. 6. This contour plot of −Δ*S*_*M*_ reveals the state of *E*_ani_, which may affect both the magnitude and the direction of magnetization vectors. Furthermore, it is noted that the magnitude of net magnetic moment is usually small but its direction is often a decisive factor when considering the exchange coupling interactions in a system^42^. According to our NPD experiment, the Tb^3+^ ions are in paramagnetic state at *T* ≥ 8 K. Therefore, the Tb-Fe(Mn) interactions should be very weak so that they can hardly be influenced by the crystal field. As the applied field increases, ZFC magnetizations in Γ_4_ phase become increasing larger, as shown by the ZFC-FC convergence near the Γ_4_−Γ_1_ transition (Fig. 5b–d). Thus, the effect of the external field plays a determining role on Γ_4_−Γ_1_ transition which to be explored in future.

**Figure 7:**
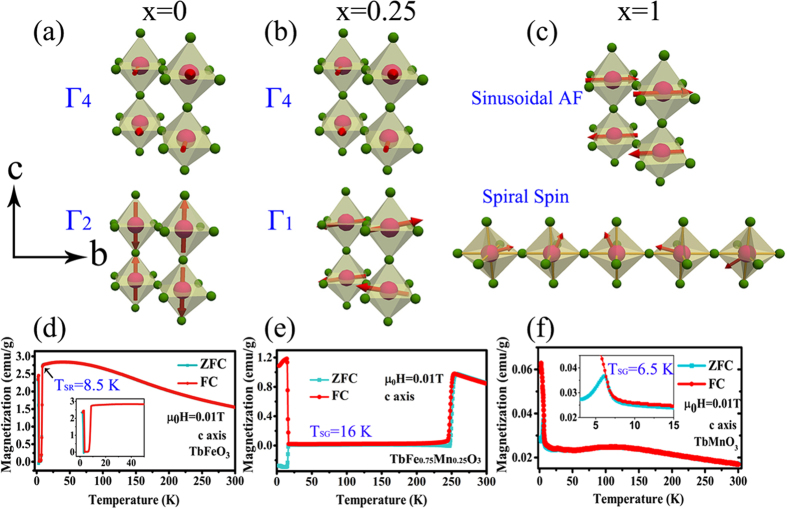
The spin configurations and spin glass state in TbFe_1−*x*_Mn_*x*_O_3_ system. (**a**) *x* = 0, no SG behavior down to 1.9 K, (**b**) *x* = 0.25, SG transition at 16 K, (**c**) *x* = 1.0, SG transition at 6.5 K. The sketches in upper panels show the variation of spin configurations in the *bc*-plane *versus x* and the lower panels show the *M*-*T* curves along the *c* axis under *H* = 100 Oe.

According to the NPD experimental results^15^,^29^, the evolution of spin configurations *versus* doping content *x* is schematically illustrated in the upper panels of Fig. 7(a–c). In the lower panels of Fig. 7(e,f), the spin glass (SG) transition *T*_SG_ occurs at 16 and 6.5 K along the *c* axis under *H* = 100 Oe in both TbFe_0.75_Mn_0.25_O_3_ and TbMnO_3_ single crystals, respectively. However, the SG state is not detected along any axis down to 1.9 K in TbFeO_3_ single crystal from Fig. 7(d), nor *a* and *b* axes in TbFe_0.75_Mn_0.25_O_3_ and TbMnO_3_ single crystals. The observation of SG behavior is attributed to the competition between AFM and FM component, leading to a spin frustration in Fig. 7(e,f). The M-O-M bond angle (M = Fe or Mn), namely, superexchange interaction angle, is usually reduced from 180° due to the cooperative octahedral rotations in the orthorhombic perovskites. In RMO_3_ systems^43^, the easy magnetization axis may rotate below *T*_N_ because of the coupling between magnetic moments of rare earth R ions and the spin of transition metal M ions. As the major controlling factor of the superexchange interaction, the coupling of M-O-M is much stronger than that of R-O-M, which should be neglected. In TbFe_1−*x*_Mn_*x*_O_3_ system, the Fe-O-Fe interaction can be partially replaced by Fe-O-Mn upon Mn^3+^ doping. In Fig. 7(b,c), the spin frustration along *c* axis causes the anisotropic superexchange interaction decreasing along the *c* axis by Mn substitution, which means the entropy change between the *a* and *c* axis gets weaker as *x* increases in a Fe-rich TbFe_1−*x*_Mn_*x*_O_3_ system. The mechanism of SG phenomena is as follows.

**Figure 8:**
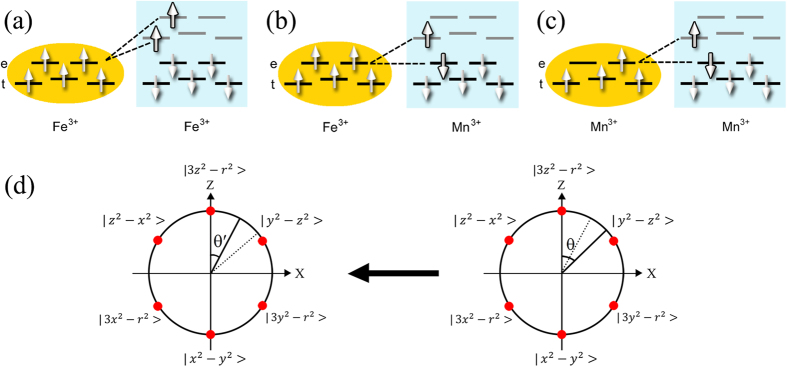
Schematic diagrams of the hybridization effect on the virtual charge transfer for (**a**) Fe-Fe, (**b**) Fe-Mn, (**c**) Mn-Mn, based on Goodenough-Kanamori rules in TbFe_1−*x*_Mn_*x*_O_3_ system. The occupied state for electrons implicates that Mn substitution promotes the FM component. (**d**) The *θ* becomes smaller when Fe doping content increases, in accompany with the weaker lattice distortion.

Since the ionic radius of Fe^3+^ is equal to Mn^3+^ in high spin state (Fe^3+^: *S* = 5/2, 5.9 *μ*_B_/at., *r* = 0.645 Å and Mn^3+^: *S* = 2, 4.9 *μ*_B_/at., *r* = 0.645 Å), the crystal distortion caused by ionic radius difference can be ignored. It should be pointed out that the hybridization between inter-site *t* and *e* orbitals is orthogonal for a 180° M-O-M chemical bonding. The sketches of Fig. 8(a–c) describe the different effects of Mn^3+^ and Fe^3+^ ions on the orbital hybridization. According to Goodenough-Kanamori rule^44^, the superexchange interaction between two adjacent transition-metal ions is delivered by a virtual charge transfer. The Fe^3+^ and Mn^3+^ ions present different configurations for the outer shell electrons, i.e., *t*^3^*e*^2^ for Fe^3+^ and *t*^3^*e*^1^ for Mn^3+^, respectively. In RFeO_3_, five outer shell electrons of Fe^3+^ ions lead to half-filled *e*_*g*_ (σ-bond component) and *t*_2g_ (π-bond component) orbitals. Therefore, the superexchange interactions between the two Fe^3+^ ions only result in an AFM coupling, in accordance to Hund’s rules. For Mn^3+^ ions, there are three kinds of coupling, i.e., *t*^3^-O-*t*^3^, *e*^1^-O-*e*^1^ and *t*^3^-O-*e*^1^. The superexchange interactions over the half-filed *t*^3^-O-*t*^3^ induce an AFM coupling. Nevertheless, the hybridization between *t*^3^-O-*e*^1^ and *e*^1^-O-*e*^1^ might provide an FM coupling in the system, which experimentally confirms that the spiral spin states in TbMnO_3_ originate from spin frustration^29^. The above argument can help us explain the SG phenomena in Fig. 7(b,c).

Now we look at the Mn-rich TbFe_1−*x*_Mn_*x*_O_3_ system. As illustrated in Fig. 8(b,c), the Mn substitution gives rise to the FM component in TbFe_1−*x*_Mn_*x*_O_3_ system with two consequences. One is the appearance of spin-glass state in Fig. 7(b,c), and the other one is the variation of anisotropic −Δ*S*_*M*_. In TbFe_1−*x*_Mn_*x*_O_3_ (*x* > 0.5), Mn^3+^ substitution can induce more FM component and lead to a stronger lattice distortion in the system. These factors induce a spin frustration state and result in the reduction of superexchange interaction. In RMnO_3_ system, the occupied *e*_*g*_ orbital wavefunction of Mn is given by Eq. (3)


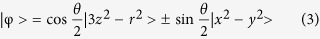


where *θ* is the respective orbital component (Fig. 8(d)). The ground state of the system is given by any normalized linear combination of the two *e*_*g*_ orbitals in Eq. (3). In TbMnO_3_, the Jahn-Teller distortion of MnO_6_ octahedral is a mode of elongating along one axis but shrinking in the other two axes. Since the rare-earth ferrites belong to Jahn-Teller inactive system, *θ* becomes smaller with doping from *x* = 1 to 0.5, and the shape of wavefunction |ϕ> will be stretched. Therefore, the distance of Mn(Fe)-Mn(Fe) along the *c* axis between the Mn(Fe)O_6_ octahedra should be longer than that in TbMnO_3._ Thus the superexchange interaction in TbFe_1−*x*_Mn_*x*_O_3_ is suppressed along the *c* axis. Additionally, the application of external magnetic field can increase the lattice distortion and enhance the DM interaction^45^,^46^. The above discussions can account for the variation of −ΔS_*M*_ for 0.5 ≤ *x* ≤ 1.

In summary, we observed a re-entrant spin reorientation of type Γ_4_ → Γ_1_ → Γ_4_ in TbFe_0.75_Mn_0.25_O_3_ perovskite system. Through neutron powder diffraction and magnetization measurements, we have observed the recurrent magnetic phase transitions at 254 and 16 K. With Mn doping, the spin configurations can be modified and the spin glass state emerges due to the competition between AFM and FM components. Furthermore, the first spin reorientation temperature increases from 8.5 K for *x* = 0 to 299 K for *x* = 0.5 TbFe_1−*x*_Mn_*x*_O_3_ sample, which might be useful for developing spin-switching devices. We have found a rich phase diagram of magnetization by tuning the applied magnetic field and temperature. In the framework of Goodenough-Kanamori rule, we analyze the evolution of −Δ*S*_*M*_ *versus* Mn doping to reveal the unusual spin reorientation and abundant magnetic phase diagram. It is found that the −Δ*S*_*M*_ decreases for 0 ≤ *x* ≤ 0.5 and increases for 0.5 ≤ *x* ≤ 1. The evolution of −Δ*S*_*M*_ is attributed to the change of anisotropic interactions tuned by Mn doping concentration. Furthermore, in an ongoing project we have discovered similar results in Mn doped HoFeO_3_ and DyFeO_3_, with Mn dopants in RFeO_3_ triggering a rare phase transition from normal Γ_4_ → Γ_2_ to re-entrant Γ_4_ → Γ_1_ → Γ_4_ spin reorientation.

## Methods

A series of Mn doped terbium orthoferrites TbFe_1−*x*_Mn_*x*_O_3_ (*x* = 0, 0.10, 0.25, 0.40, 0.50, 0.60, 0.70, 0.80, 0.90, 1) polycrystalline samples were first synthesized by traditional solid state reaction method. Single crystals of *x* = 0, 0.10, 0.25 and 1 were then grown by using a four-mirror optical floating-zone furnace (FZ-T-10000-H-VI-P-SH, Crystal System Corp.). The phase purity, crystal quality, and crystallographic orientation were checked by powder X-ray diffraction (XRD) and back-reflection Laue XRD experiment, respectively. Magnetization measurements were performed on vibrating sample magnetometer (VSM) attached to a physical property measurement system (PPMS-9), and both zero-field-cooling (ZFC) and field-cooling (FC) modes were used. The NPD experiments at 8, 40, and 300 K were carried out on the thermal triple-axis spectrometer SV30 located at China Advanced Research Reactor (CARR) in China Institute of Atomic Energy, and the neutron powder diffractor at the Institute of Nuclear Physics and Chemistry, China Academic of Engineering Physics. The structural data analyses of XRD and NPD were performed by FullProf program using Rietveld method^47^, and the magnetic symmetry analysis was performed with BasIReps.

## Additional Information

**How to cite this article**: Fang, Y. *et al.* Observation of re-entrant spin reorientation in TbFe_1−x_Mn_x_O_3_. *Sci. Rep.* **6**, 33448; doi: 10.1038/srep33448 (2016).

## Supplementary Material

Supplementary Information
