# Supplementary material for: Observation of re-entrant spin reorientation in TbFe1−xMnxO3
Source: Sci Rep. 2016 Sep 16;6:33448. doi: 10.1038/srep33448 (PMC5025771; doi:10.1038/srep33448)
Supplement: Supplementary Information [file srep33448-s1.pdf]

Supplemental information for Observation of re-entrant spin reorientation in $TbFe_{1-x}Mn_xO_3$

Yifei Fang[1,2], Ya Yang[2], Xinzhi Liu[3], Jian Kang[2], Lijie Hao[3], Xiping Chen[4], Lei Xie[4], Guangai Sun[4], Venkatesh Chandragiri[2], Chin-Wei Wang[6], Yiming Cao[2], Fei Chen[1], Yuntao Liu[3], Dongfeng Chen[3], Shixun Cao[1,2], Chengtian Lin[5], Wei Ren[2*] and Jincang Zhang[1,2*]

[1] Materials Genome Institute, Shanghai University, Shanghai 200444, China

[2] Department of Physic, Shanghai University, Shanghai 200444, China

[3] Neutron Scattering Laboratory, China Institute of Atomic Energy, Beijing 102413, China

[4] Insititute of Nuclear Physics and Chemistry, China Academy of Engineering Physics, Mianyang 621999, China

[5] Max Planck Institute for Solid State Research, Heisenbergstraße 1, D-70569 Stuttgart, Germany

[6] ANSTO, Kirrawee Dc, NSW 2232, Australia

Corresponding Authors, E-mails: renwei@shu.edu.cn; jczhang@shu.edu.cn

**8 K**	*a*	*b*	*c*
Fe/Mn1 (A-type)	3.404	0.616	0.840
Fe/Mn2 (B-type)	-3.404	-0.616	0.840
Fe/Mn3 (C-type)	-3.404	0.616	0.840
Fe/Mn4 (D-type)	3.404	-0.616	0.840
**40 K**	*a*	*b*	*c*
Fe/Mn1 (A-type)	0.712	3.724	0.636
Fe/Mn2 (B-type)	-0.712	-3.724	0.636
Fe/Mn3 (C-type)	0.712	-3.724	-0.636
Fe/Mn4 (D-type)	-0.712	3.724	-0.636
**300 K**	*a*	*b*	*c*
Fe/Mn1 (A-type)	3.308	0.628	0.164

Fe/Mn2 (B-type)	-3.308	-0.628	0.164
Fe/Mn3 (C-type)	-3.308	0.628	0.164
Fe/Mn4 (D-type)	3.308	-0.628	0.164

Table 1 Magnetic moments along different crystallographic axes obtained from neutron powder diffraction for $TbFe_{1-x}Mn_xO_3$ (x=0.25) single crystal.
